# Characterization of Bioactivities and Biosynthesis of Angucycline/Angucyclinone Derivatives Derived from *Gephyromycinifex aptenodytis* gen. nov., sp. nov.

**DOI:** 10.3390/md20010034

**Published:** 2021-12-29

**Authors:** Wen-Zhuo Zhu, Shu-Heng Wang, Hui-Min Gao, Ya-Ming Ge, Jun Dai, Xiao-Ling Zhang, Qiao Yang

**Affiliations:** 1 Department of Marine Chemistry, College of Marine Science and Technology, Zhejiang Ocean University, Zhoushan 316022, China; zhuwenzhuo@zjou.edu.cn (W.-Z.Z.); wangshuheng@zjou.edu.cn (S.-H.W.); gaohuimin@zjou.edu.cn (H.-M.G.); 2 National Engineering Research Center for Marine Aquaculture, Zhejiang Ocean University, Zhoushan 316021, China; geyaming@zjou.edu.cn; 3 Natural “111” Center for Cellular Regulation and Molecular Pharmaceutics, Key Laboratory of Fermentation Engineering (Ministry of Education), College of Bioengineering, Hubei University of Technology, Wuhan 430068, China; jundai@hbut.edu.cn; 4 ABI Group, Zhejiang Ocean University, Zhoushan 316022, China; 5 Department of Environment Science and Engineering, Zhejiang Ocean University, Zhoushan 316022, China

**Keywords:** gut microbiota, *Gephyromycinifex aptenodytis*, angucycline/angucyclinone derivatives, exopolysaccharides, type III PKS, biogenetic pathway, Antarctic actinobacteria

## Abstract

Strain NJES-13^T^ is the type strain and currently the only species of the newly established actinobacteria genera *Aptenodytes* in the family *Dermatophilaceae* isolated from the gut microbiota of the Antarctic emperor penguin. This strain demonstrated excellent bioflocculation activity with bacteria-derived exopolysaccharides (EPSs). Moreover, it produced bioactive angucycline/angucyclinone derivatives (ADs) and contained one type III polyketide synthase (T3PKS), thus demonstrating great potential to produce novel bioactive compounds. However, the low productivity of the potential new AD metabolite was the main obstacle for its chemical structure elucidation. In this study, to increase the concentration of targeted metabolites, the influence of cellular morphology on AD metabolism in strain NJES-13^T^ was determined using glass bead-enhanced fermentation. Based on the cellular ultra-structural observation driven by bacterial EPSs, and quantitative analysis of the targeted metabolites, the successful increasing of the productivity of three AD metabolites was achieved. Afterward, a new frigocyclinone analogue was isolated and then identified as 2-hydroxy-frigocyclinone, as well as two other known ADs named 2-hydroxy-tetrangomycin (2-HT) and gephyromycin (GPM). Three AD metabolites were found to demonstrate different bioactivities. Both C-2 hydroxyl substitutes, 2-hydroxy-tetrangomycin and 2-hydroxy-frigocyclinone, exhibited variable inhibitory activities against *Staphylococcus aureus*, *Bacillus subtilis* and *Candida albicans*. Moreover, the newly identified 2-hydroxy-frigocyclinone also showed significant cytotoxicity against three tested human-derived cancerous cell lines (HL-60, Bel-7402 and A549), with all obtained IC_50_ values less than 10 µM. Based on the genetic analysis after genomic mining, the plausible biogenetic pathway of the three bioactive ADs in strain NJES-13^T^ was also proposed.

## 1. Introduction

*Actinobacteria* are unique prokaryote groups and virtually unlimited sources due to their extraordinary abilities to deliver a multitude of novel bioactive natural products with a variety of medical, pharmaceutical, industrial and ecological significance [1,2]. Among the *Actinobacteria*, the genus *Streptomyces* has the most abundant bacterial diversity, containing 682 validly published type species (https://lpsn.dsmz.de/genus/streptomyces, assessed on 25 December 2021). These valuable actinobacteria are recognized as the most notable bacterial group for natural product production, with their considerable diversity in chemical structures and bioactivities implying a variety of pharmaceutical applications [3]. Of these, the polyketide-derived metabolites are among the most diverse group and include many clinically important natural compounds [4].

The angucycline/angucyclinone (sugarless) derivatives (ADs), which are aromatic polyketides with a tetracyclic benz[a]anthracene skeleton, include the largest group of polyketide synthase (PKS)-engineered natural products and comprise a rapidly growing class of bioactive secondary metabolites derived from a variety of microbial sources [5,6,7]. Among plenty of ADs, gephyromycin, which was originally isolated from *Streptomyces griseus* in 2005, is the first representative angucyclinone with an unprecedented intramolecular ether bridge [8]. Gephyromycin is one vital group of angucyclinones, which have been found to have over 100 derivatives since their first discovery in 1965 [9] and have attracted much attention due to their broad range of bioactivities, mainly as potential antiviral, antimicrobial, anti-tumor and enzyme inhibitory drugs [5,10,11,12]. Previous studies have shown that gephyromycin A exhibits powerful glutaminergic activity toward neuronal cells with a comparable effective dosage against DCG-IV, which is the most potent glutamate agonist [8]. Gephyromycin C demonstrates obvious anti-tumor effects toward human prostate cancer PC3 cells (IC_50_ = 1.38 µmol/L) as a novel small-molecule HSP90 inhibitor [10].

For the bacteria-producing sources of gephyromycins, they only covered a single actinobacteria division, *Streptomyces* sp. These natural producers include Antarctic territorial strain NTK 14 [8], strain M268 isolated from marine sediment producing gephyromycin A [13], marine strain SS13I producing gephyromycins B and C [14] and *Streptomyces* sp. strain HDN15129 producing *ent*-gephyromycin A and five other monacycliones G–K [15]. However, none of the gephyromycins had been reported in bacteria other than *Streptomyces* members until our previous discovery [11]. Recently, we reported the isolation of a novel actinobacterium strain, NJES-13^T^, from the gut microbiota of the Antarctic emperor penguin *Aptenodytes forsteri* [12]. The new bacterial isolate was found to produce two known angucyclinone derivatives, named gephyromycin (GPM) and 2-hydroxy-tetrangomycin (2-HT), as well as complex active bioflocculation exopolysaccharide (EPS) metabolites [16,17,18]. Moreover, one extra potential new angucycline derivative was also detected, although its chemical structure was not elucidated due to its native low production level [11]. Strain NJES-13^T^ is the type strain and the only species of the newly established genera *Aptenodytes* in the family *Dermatophilaceae* proposed by our previous polyphasic taxonomic characterization [11]. Moreover, strain NJES-13^T^ was found to contain a series of biosynthesis gene clusters (BGCs), including one type III polyketide synthase (T3PKS) cluster, which was possibly responsible for the biosynthesis of AD metabolites based on bioinformatic analysis of the complete genome sequence of strain NJES-13^T^ [11,12].

In this study, the previously assumed new angucycline derivative was isolated and identified as 2-hydroxy-frigocyclinone from the metabolites of strain NJES-13^T^, together with known 2-hydroxytetrangomycin and gephyromycin after the cultivation based on the designed glass bead-enhanced fermentation, which successfully increased the productivity levels of targeted AD metabolites in this filamentous actinobacteria strain. Moreover, three AD metabolites exhibited different bioactivities. Both C-2 hydroxyl substitutes exhibited various inhibitory activity against *Staphylococcus aureus*, *Bacillus subtilis* and *Candida albicans*. Moreover, 2-hydroxy-frigocyclinone also demonstrated significant cytotoxicity against three tested human-derived cancerous cell lines (HL-60, Bel-7402 and A549), with all obtained IC_50_ values less than 10 µM. Based on the genetic analysis by genomic mining, the plausible biogenetic pathway of the three AD metabolites derived from strain NJES-13^T^ was also proposed.

## 2. Results and Discussion

### 2.1. Fermentation Based on Glass Bead-Enhanced Cultivation of Strain NJES-13^T^

**Figure 1:**
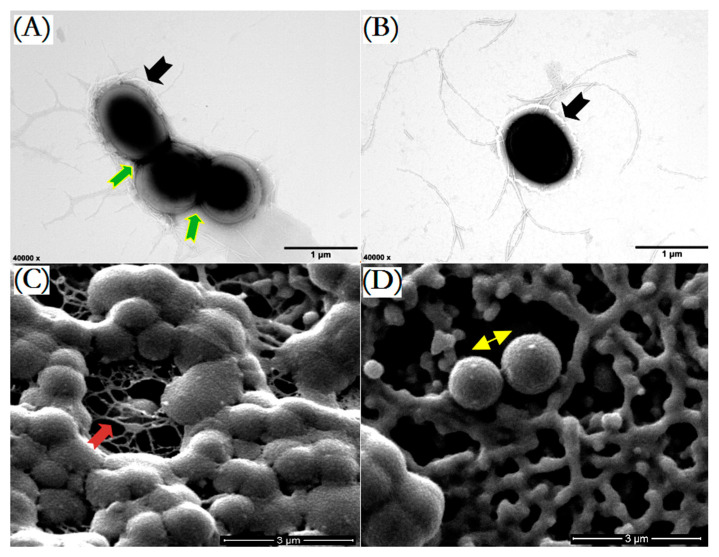
Cellular morphological characteristics of strain NJES-13^T^ after 10 days cultivation at 28 °C in R_2_A broth. Transmission electron microscopy (TEM) observation showed the coccoid- shaped cells with clusters surrounded by a slimy outermost extracellular layer composed of bacteria-derived exopolysaccharides (EPSs) [black arrows in (**A**,**B**)] and the cell aggregation (green arrows in (**A**). Scanning electron microscopy (SEM) observation revealed that the cell clusters were interconnected by viscous EPSs and showed a dimensional 3D net-like morphology [red arrow in (**C**)], and the partly interrupted and separated cells [yellow arrow in (**D**)] after applying the glass bead-enhanced cultivation were also observed. *Bar*, 1 μm for (**A**,**B**) and 3 μm for (**C**,**D**).

Previously, two angucyclinone derivatives were isolated and identified from the fermentation metabolites of strain NJES-13^T^ [11]. Moreover, one extra major HPLC peak was also found and indicated the presence of a potential new AD metabolite (later identified as 2-hydroxy-frigocyclinone) in fermentation products, but the low productivity did not allow for enough compound to be obtained for chemical structure elucidation [11]. For the actinomycete, cellular morphology is regarded as one key aspect in understanding the secondary metabolism in filamentous groups [19,20,21]. Based on ultra-structural observations of the single and aggregated cells of strain NJES-13^T^ using a transmission electron microscope (TEM), strain NJES-13^T^ formed rod-shaped cells with the filaments, surrounded by a slimy outside extracellular layer mainly composed of bacteria-derived macromolecular exopolysaccharides (EPSs) (Figure 1A,B), which is regarded as the main essential chemical basis for host–bacteria interactions [16,17,18,22]. Moreover, based on scanning electron microscope (SEM) observations, the cells of strain NJES-13^T^ were found to be clustered and interconnected by viscous EPSs showing an unusual three-dimensional and net-like morphology (Figure 1C).

**Figure 2:**
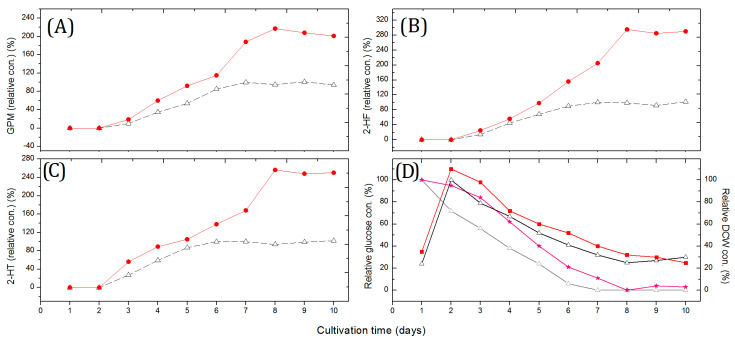
The relative concentrations (con., %) of the three angucycline/angucyclinone derivative (AD) metabolites, namely, (**A**) gephyromycin (GPM), (**B**) 2-hydroxy-tetrangomycin (2-HT) and (**C**) 2-hydroxy-frigocyclinone (2-HF), and (**D**) the glucose and dry cell weight (DCW) of a 10-day-long cultivation time for flask fermentation of strain NJES-13^T^ with (solid line with circle) and without (dotted line with triangle) glass bead addition (Ø = 500 μm, 100 g/L).

The addition of micro- or macro-particles has been proven to be beneficial for many filamentous microorganisms to promote their secondary metabolite formation [21,23,24]. To determine whether the cellular morphology influences AD metabolism in the filamentous actinobacteria strain NJES-13^T^, the production levels of three AD metabolites were measured and compared with the addition of a variety of sizes of glass beads (Ø, 100–2000 μm) at a concentration of 100 g/L into the cultivation system of strain NJES-13^T^ in 500 mL baffled shaking flasks. The obtained results showed that the productivity of the three AD metabolites all reached the maximum level with the addition of 500 μm beads. With 500 μm beads added into the cultivation flasks, a 2.78-fold increase in 2-HF concentration was achieved compared to the control (Figure 2), as well as enhancing effects on the production of the other two known AD metabolites. Based on TEM observation, the clustered cells of strain NJES-13^T^ were found to be partly interrupted (Figure 1B). This change in cell aggregation was also confirmed by SEM observation; the state of mycelium in balls or clumps in the untreated group was much more improved by the applied glass bead-enhanced cultivation (Figure 1D). The mechanism of action may be explained by the mechanical stress induced by the glass beads, which is often considered to be responsible for the effect on cell morphology, since mechanical stress by aeration or agitation can also influence pellet morphology [21,24].

### 2.2. New Angucyclinone Derivative Metabolite Characterization

**Figure 3:**
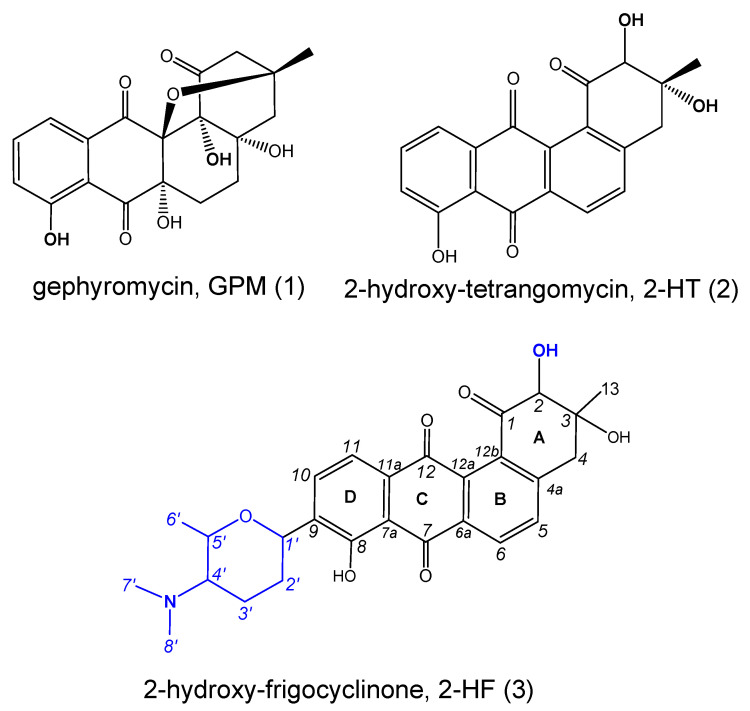
Chemical structures of the three AD metabolites gephyromycin (GPM, **1**), 2-hydroxy-tetrangomycin (2-HT, **2**) and 2-hydroxy-frigocyclinone (2-HF, **3**) derived from strain NJES-13^T^.

**Figure 4:**
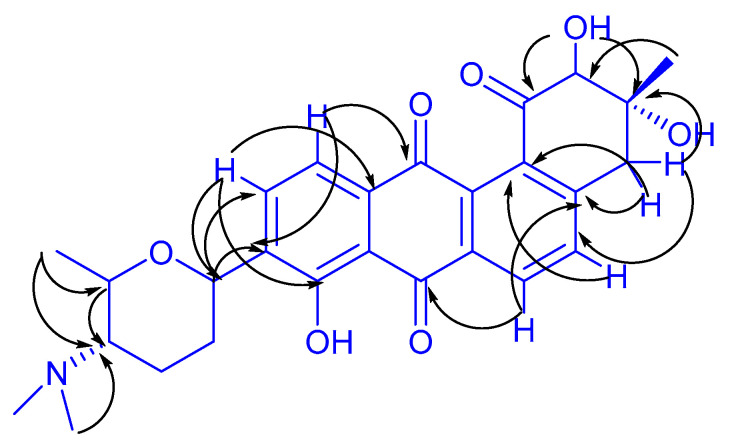
Key HMBC (H→C) correlation analysis of 2-HF.

With the aid of glass bead-enhanced fermentation cultivation and the greatly increasing productivity of the ADs metabolites in strain NJES-13^T^, the third unidentified AD (**3**) was isolated, along with two other known ADs named gephyromycin (**1**, GPM) and 2-hydroxy-tetrangomycin (**2**, 2-HT) as shown in Figure 3, and the new AD’s structure was subjected to a detailed chemical elucidation. High-resolution electrospray ionization mass spectroscopy (HR-ESI-MS) showed a pseudomolecular ion at *m*/*z* 480.2019 [M + H]^+^, suggesting molecular formula C_27_H_29_NO_7_ (calculated value: 480.2017) (Figure S1). In addition, ^1^H and ^13^C NMR analyses (Table 1 and Figure S1) showed similar characteristics to frigocyclinone, a known angucyclinone derivative with an unusual C-glycosidic-linked aminodeoxy sugar moiety attached to an anthraquinone chromophore, which was originally isolated from an Antarctica territorial species of *Streptomyces griseus* [25]. The detailed structure of this metabolite produced by strain NJES-13^T^ was finally elucidated as a new frigocyclinone analogue, named 2-hydroxy- frigocyclinone (**3**, 2-HF, Figure 3), by the chemical characterization evidence obtained by the HSQC (Figure S1) and HMBC correlation analyses (Figure 4 and Figure S1).

### 2.3. Bioactivity Assays of Three AD Metabolites

After the preparation of the three AD metabolites, they were evaluated for antimicrobial and cytotoxic activities. Minimum inhibitory concentration (MIC) studies were performed according to the standard reference method [26]. The obtained results are shown in Table 2. It can be seen that both C-2 hydroxyl substitutes, 2-hydroxy-tetrangomycin and 2-hydroxy-frigocyclinone, exhibited various inhibitory activities against *Bacillus subtilis* ATCC 6051, *Staphylococcus* *aureus* ATCC 12600 and *Candida* *albicans* ATCC 10231. However, gephyromycin exhibited no obvious inhibitory effect on the three test bacterial strains. For the cytotoxic activity evaluation, three human-derived cancerous cell lines (HL-60, Bel-7402 and A549) were used to study the anti-tumor effects of the three AD metabolites. Among the three ADs, 2-hydroxy-frigocyclinone showed significant cytotoxicity against the three tested cell lines, with all IC_50_ values less than 10 µM. 2-hydroxy-frigocyclinone exhibited moderate cytotoxicity, with IC_50_ values ranging from 14.1 to 25.8 µM. However, gephyromycin showed no obvious cytotoxicity against the three cells.

### 2.4. Biosynthetic Gene Analysis

According to the prediction analysis of biosynthetic gene clusters (BGCs) responsible for secondary metabolite synthesis using the antiSMASH tool [27,28,29], five BGCs were found in the genome of strain NJES-13^T^. This included one 44.5-kb-long type III polyketide synthase (T3PKS) cluster, one 47.4-kb-long type I polyketide synthase (T1PKS) cluster, two NRPS-like clusters that were 43.4 kb and 42.0 kb long and one 27.8-kb-long beta-lactone cluster. These findings indicate that strain NJES-13^T^ demonstrates great potential to produce new bioactive metabolites [30].

**Figure 5:**
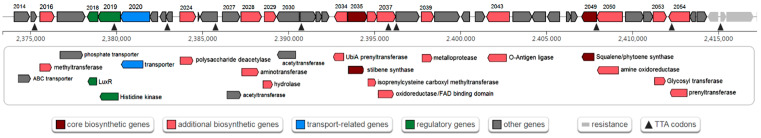
Schematic diagram of the gene cluster responsible for the biosynthesis of the AD metabolites derived from strain NJES-13^T^.

Comparative bioinformatics analysis revealed that the gene locus ctg1_2030 (1320 nt) embraces three domains, including two acetyltransferases (ATs) and one sterol carrier protein (SCP) domain, and suggested the same strategy to generate the propionyl-CoA starter unit used in strain NJES-13^T^ (Figure 5). Additionally, a cluster of potential core biosynthesis genes, stilbene synthase (STS) genes (ctg1_2035), occurred within an approximately 2373–2418 kb region on the chromosome of strain NJES-13^T^, showing 100% similarity to the alkylresorcinol biosynthetic gene cluster from the streptomycin-producing *Streptomyces griseus* subsp. *griseus* NBRC 13350 [31]. Upstream and downstream of this core region, UbiA prenyltransferase (ctg1_2034), isoprenylcysteine carboxyl methyltransferase (LOC_2036) and FAD-dependent oxygenase (ctg1_2037) were also included, which were predicted to encode type III PKS-like polypeptides and shared approximately 91.7, 95.6 and 92.1% amino acid identities, respectively. Additional biosynthetic genes, including methyltransferase (LOC_2016), polysaccharide deacetylase (LOC_2024), aminotransferase (LOC_2028), amine oxidoreductase (LOC_2050) and glycosyltransferase (ctg1_2053), were also found in the locus among the genome of strain NJES-13^T^ as shown in Figure 5.

### 2.5. Proposed Biogenetic Pathway for Three AD Metabolites

**Figure 6:**
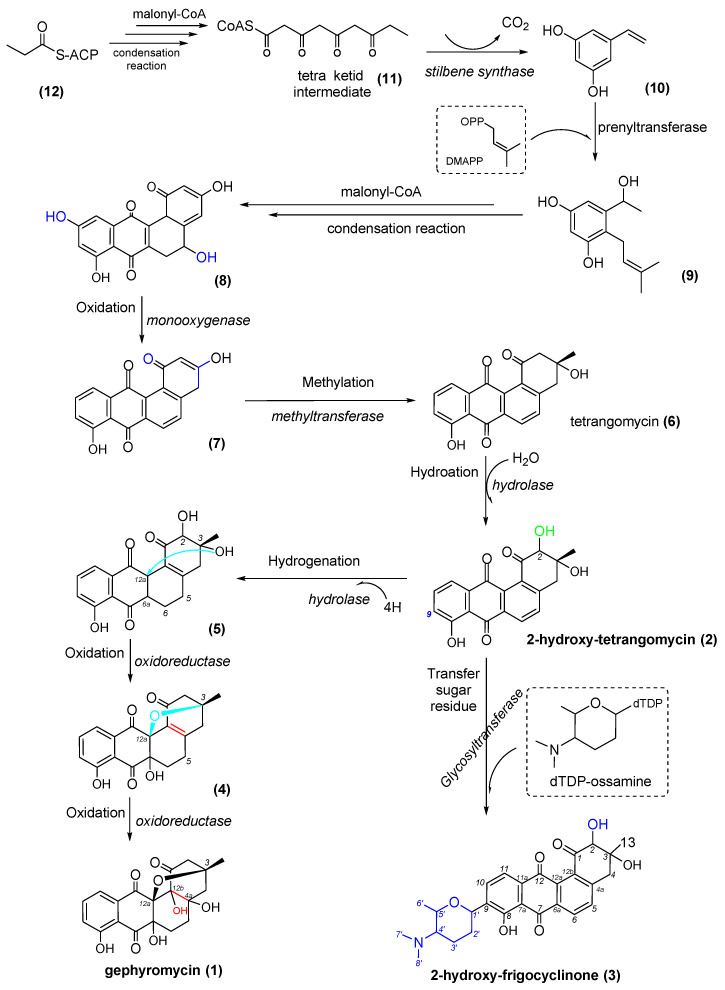
Plausible biogenetic pathway of the three ADs metabolites in strain NJES-13^T^.

Based on the bioinformatics analysis of the genomic feature, a plausible biogenetic pathway of the three AD metabolites in strain NJES-13^T^ was proposed, and the obtained result is presented in Figure 6. The typical type III PKS gene cluster utilized acyl-CoA (**12**) as the starter unit, performed three condensation reactions with malonyl-CoA as the extender unit and yielded a tetraketide intermediate (**11**). Moreover, a following ring-folding reaction was achieved with aldol condensation to afford the stilbene-like intermediate (**10**) [32]. Then, 10 was converted to form alkylresorcinol (**9**) catalyzed by prenyltransferase (ctg1_2034 and ctg1_2054) in the presence of dimethylallyl pyrophosphate (DMAPP) and through a furthering condensation reaction to yield the early intermediate (**8**). Following this, 8 was taken to several post-PKS tailoring steps with the involvement of oxidoreductase (ctg1_2037). The gene locus ctg1_2037 was predicted to be a FAD-dependent bifunctional oxygenase/reductase with an N-terminal flavin-dependent oxygenase domain and a C-terminal reductase domain, which belongs to the short-chain dehydrogenase/reductase (SDR) superfamily [33]. The gene locus (ctg1_2037) was predicted to be involved in the transformation from **8** to intermediate **7** and then to aglycone tetrangomycin (**6**). At this stage, tetrangomycin was proposed to be transformed into 2-hydro-tetrangomycin (**2**) through the hydrogenation reaction that occurred at the C-2 position catalyzed by the hydrolase (ctg1_2029), which was regarded as the main intermediate reaction in the angucycline/angucyclinone derivative biosynthetic pathway in strain NJES-13^T^. 2-hydro-tetrangomycin (**2**) was supposed to undergo two steps of Baeyer–Villiger oxygenation catalyzed by the bifunctional oxygenase/reductase (ctg1_2037) to form the two intermediate products, **5** plus **4**, and, finally, to form gephyromycin (**1**). In a separate shunt pathway, the formation of 2-hydro-frigocyclinone (**3**) was likely to be achieved from 2-hydro-tetrangomycin (**2**) through C-9 glycosylation catalyzed by glycosyltransferase (ctg1_2053), which was proposed to be responsible for the formation of the C-glycosidic linkage between ossamine and tetrangomycin [25,34]. However, further efforts are still required to verify the genetic details and, thus, map out the biosynthetic pathway in strain NJES-13^T^.

## 3. Materials and Methods

### 3.1. Bacterial Culture

Strain NJES-13^T^ was routinely cultured on R_2_A agar or broth (Difco, BD, Franklin Lakes, NJ, USA), cultivated for 10 days at 28 °C, cultivated on the same medium in slant tubes, maintained as a glycerol suspension (30%, *v*/*v*) and stored at −80 °C for long-term preservation [35,36].

### 3.2. Glass Bead-Enhanced Fermentation

The inoculation of the main culture of strain NJES-13^T^ was conducted by transferring 500 μL of the pre-culture into each shaking flask. Baffled shaking flasks with a total volume of 500 mL filled with 100 mL R_2_A medium were used. The main cultures were incubated with continuous shaking at 200 rpm on an orbital shaker (Model 5000I, VWR, Radnor, PA, USA), cultured at 28 °C for 10 days as previously described [11] for the pre-culture and sampled daily. For the enhancing experiments, glass beads (100 g/L, with diameters ranging from 100 to 2000 μm, Merck, Darmstadt, Germany) were added to the culture media to test the potential effects on the productivity of the active metabolites [37].

### 3.3. Bacterial Metabolite Isolation

A total of 1 L of fermentation broth was harvested and filtered. The harvested cells were extracted with cold methanol twice [11]. The combined extracts were concentrated to about 100 mL volume under reduced pressure, then subjected to the MCI silica column (Mitsubishi Chemical, Tokyo, Japan) and eluted with gradient MeOH/H_2_O solvent (10:90, 50:50, 80:20 and 100, *v*/*v*) to obtain three elution fractions. The 80% MeOH fraction was separated by Sephadex LH-20 gel column (2.5 × 75 cm) and then eluted with ethanol to obtain two fractions. The purification procedure was performed on a semi-preparative RP-HPLC (C_18_, 10 mm × 250 mm, 5 μm) using a gradient elution ranging from 0 to 100% MeOH/H_2_O solvents to obtain pure compounds **1** (GPM) and **3** (2-HF) and for the other fraction to obtain pure compound **2** (2-HT).

### 3.4. Chemical Structure Characterization

The ^1^H (400 MHz) and ^13^C NMR (101 MHz) spectra were recorded with a Bruker AM-400 spectrometer (Bruker Daltonics, MA, USA) using tetramethylsilane (TMS) as the internal standard. ESI-HRMS was performed on a Waters Micromass LCT Premier mass spectrometer connected with an Agilent 1200 HPLC system (Waters, Milford, MA, USA) [11,12,13,14,15].

### 3.5. Quantitative Analysis of Metabolites

For HPLC quantitative analysis of the target metabolites, we used an Agilent 1200 HPLC system (Agilent, Santa Clara, CA, USA) equipped with an analytical Hypersil ODS column (5 μm, 250 × 4.6 mm, Thermo Fisher Scientific, Waltham, MA, USA) and operated at 28 °C. A gradient elution using increasing concentrations of MeOH in CH2Cl2 was used with a constant flow rate of 1 mL/min. The detection monitored by diode array detector was set at the wavelength of 320 nm.

### 3.6. Glucose and Dry Cell Weight Concentration Measurements

Glucose concentrations during fermentation were determined using an Agilent 1100 HPLC system equipped with a refractive index (RI) detector using pure water for elution at a constant flow rate of 0.7 mL/min. The Agilent Metacarb 87C analytical column (300 × 7.8 mm, Agilent, Santa Clara, CA, USA) operated at 85 °C was used. The dry cell weights (DCWs) of the bacterial biomass were gravimetrically determined using 2 mL of the fermentation samples and conducted in duplicates.

### 3.7. Bioactivity Evaluations

The antimicrobial activity was determined against two Gram-positive bacterial strains, namely, *Staphylococcus aureus* ATCC 12600 and *Bacillus subtilis* ATCC 6051, and one yeast *Candida albicans* ATCC 10231 using standard microplate assays [37,38]. For cytotoxic activity assay, three human-derived cancerous cell lines, namely, human leukemic cell line HL-60, human hepatocellular carcinoma cell line Bel-7402 and human lung cancer cell line A549, were used according to a standard MTT method [39] and performed in triplicate in a microplate reader. IC_50_ value was taken using SPSS 17.0 software. All the results are expressed as means ± SD. The statistical significance was analyzed using *t*-test in SPSS Statistics (version 17.0) and plotted with Origin (version 8.0) [40,41,42,43]. A value of *p* < 0.05 was considered statistically significant.

### 3.8. Biosynthetic Pathway Analysis of ADs

The prediction of the biosynthetic gene clusters (BGCs) for secondary metabolite synthesis was performed using the Secondary Metabolite Analysis Shell (antiSMASH) version 6.0.1 [27,44,45,46] based on the whole genome sequence of strain NJES-13^T^ [11]. The genome sequence data were also analyzed by Blast2GO tool [47,48] to help to predict the potential cluster gene functions and to confirm genetic functions by manual BLASTx/BLASTp searching in NCBI non-redundant (NR) protein sequence database.

## 4. Conclusions

Actinobacteria are enormously important in pharmaceutical, industrial and ecological applications due to their unlimited ability to produce various natural drugs, enzymes and bioactive natural products. The significant diversity of rare actinomycetes inhabiting gut microbiota and their potential capacity to synthesize a diverse reservoir of valuable bioactive metabolites, as well as them being key actors interacting with the host and other organisms, have been well demonstrated. In this study, the combined bioactivity and biosynthesis characterizations of the type strain NJES-13^T^ isolated from the gut microbiota of the Antarctic emperor penguin clearly demonstrated its great potential as a fresh actinobacterial candidate, representing a valuable source to deliver a series of biologically active angucycline/angucyclinone derivative metabolites with significant antimicrobial and cytotoxic activities, as well as polyketide synthase (PKS)-driven genes for their biosynthesis. Further characterizations, including structure–activity relationship (SAR) analysis and molecular mechanism, are vital to clarify the biotechnological importance and ecological roles of this type species within the newly discovered actinomycete genera.

## Figures and Tables

**Table 1:** The ^1^H and ^13^C NMR data for 2-hydroxy-frigocyclinone (2-HF).

Position	*δ*_H_	*δ*_C_	Position	*δ*_H_	*δ*_C_
1	—	192.5, Cq	12	—	172.1, Cq
2	4.90	75.2, CH	12a	—	138.2, Cq
3	—	73.2, Cq	12b	—	139.1, Cq
4	2.63	42.2, CH_2_	13	1.32 (s)	20.3, CH_3_
4a	—	147.2, Cq	1’	4.92_ax_ (m)	78.7, CH
5	7.57	132.6, CH	2’	2.05_eq_/1.45_ax_ (m)	30.7, CH_2_
6	7.92 (d, 8.0)	129.9, CH	3’	1.84_eq_/1.58_ax_ (m)	20.2, CH_2_
6a	—	132.5, Cq	4’	2.25_ax_ (m)	72.4, CH
7	—	181.0, Cq	5’	3.98_eq_ (m)	78.5, CH
7a	—	118.5, Cq	6’	1.21 (d, 6.8)	10.7, CH_3_
8	—	159.9, Cq	7’, 8’	2.20 (s)	41.8, CH_3_
9	—	138.4, Cq	2-OH	6.04 (s)	—
10	7.40	132.4, CH	3-OH	4.95 (br)	—
11	7.43	120.7, CH	8-OH	12.10 (s, br)	—
11a	—	136.2, Cq			

*d* from TMS in DMSO-*d*_6_; *J* in Hz.

**Table 2:** The bioactivity evaluation results of the three AD metabolites derived from strain NJES-13^T^.

Tested Compounds	Antibacterial Activity (MIC, µg/mL)			Cytotoxic Activity (IC_50_, µM)		
	*B. subtilis*	*S. aureus*	*C. albicans*	HL-60	Bel-7402	A549
Gephyromycin (GPM)	>100	>100	>100	133.2	108.7	154.3
2-Hydroxy-tetrangomycin (2-HT)	27.2	14.1	15.6	25.8	35.6	14.1
2-Hydroxy-frigocyclinone (2-HF)	15.4	5.7	8.5	8.4	4.2	5.5

MIC, minimum inhibitory concentration; IC_50_, half maximal inhibitory concentration; HL-60, human leukemic cell line; Bel-7402, human hepatocellular carcinoma cell line; A549, human lung cancer cell line; *S. aureus*, *Staphylococcus aureus* ATCC 12600; *B. subtilis*, *Bacillus subtilis* ATCC 6051; *C. albicans*, *Candida albicans* ATCC 10231.

## Data Availability

The article contains all the data produced in this study.

## Supplementary Materials

The following supporting information can be downloaded at: https://www.mdpi.com/article/10.3390/md20010034/s1; Figure S1: The NMR spectra data of 2-hydroxy-frigocyclinone (2-HF).
