# Supplementary material for: Characterization of Bioactivities and Biosynthesis of Angucycline/Angucyclinone Derivatives Derived from Gephyromycinifex aptenodytis gen. nov., sp. nov
Source: Mar Drugs. 2021 Dec 29;20(1):34. doi: 10.3390/md20010034 (PMC8779670; doi:10.3390/md20010034)
Supplement: Supplementary file 1 [file marinedrugs-20-00034-s001.zip › marinedrugs-1528551-supplementary.pdf]

Supplementary Materials

Characterization of Bioactivities and Biosynthesis of Angucycline/Angucyclinone Derivatives Derived from *Gephyromycinifex aptenodytis* gen. nov., sp. nov.

Wen-Zhuo Zhu [1], Shu-Heng Wang [1], Hui-Min Gao [1], Ya-Ming Ge [2], Jun Dai [3], Xiao-Ling Zhang [1,4,*] and Qiao Yang [1,4,5,*]

[1] Department of Marine Chemistry, College of Marine Science and Technology, Zhejiang Ocean University, Zhoushan 316022, China; zhuwenzhuo@zjou.edu.cn (W.-Z.Z.); wangshuheng@zjou.edu.cn (S.-H.W.); gaohuimin@zjou.edu.cn (H.-M.G.)

[2] National Engineering Research Center for Marine Aquaculture, Zhejiang Ocean University, Zhoushan 316021, China; geyaming@zjou.edu.cn

[3] Natural “111” Center for Cellular Regulation and Molecular Pharmaceutics, Key Laboratory of Fermentation Engineering (Ministry of Education), College of Bioengineering, Hubei University of Technology, Wuhan 430068, China; jundai@hbut.edu.cn

[4] ABI Group, Zhejiang Ocean University, Zhoushan 316022, China

[5] Department of Environment Science and Engineering, Zhejiang Ocean University, Zhoushan 316022, China

* Correspondence: zhangxiaoling@zjou.edu.cn (X.-L.Z.); qiaoyang1979@whu.edu.cn (Q.Y.)

Figure S1. The NMR spectra data of 2-hydroxy-frigocyclinone (2-HF).

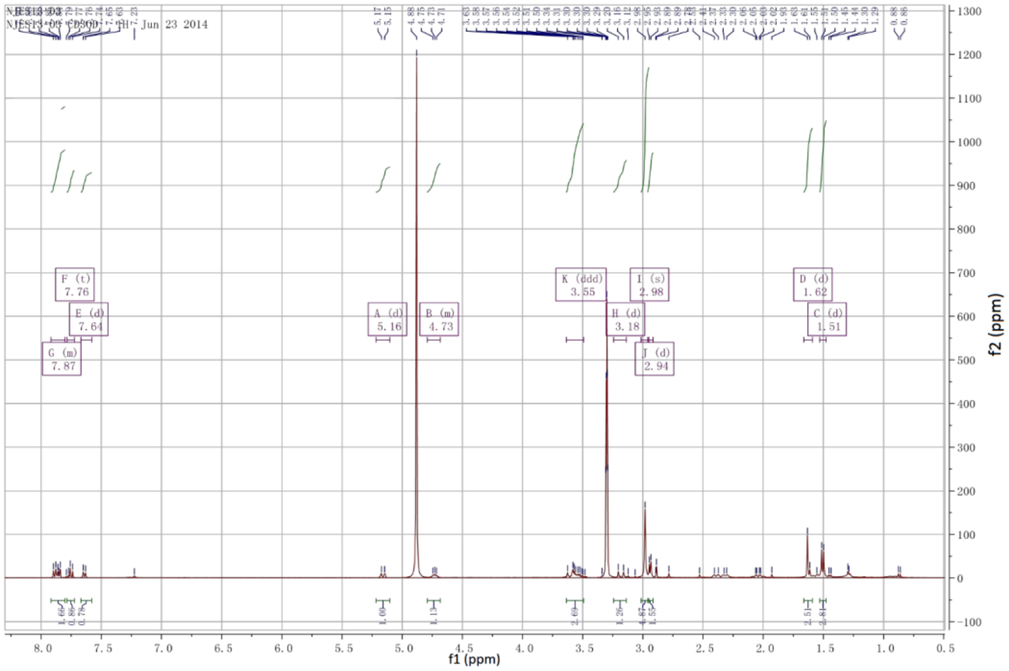

(1) ^{1}H NMR spectrum

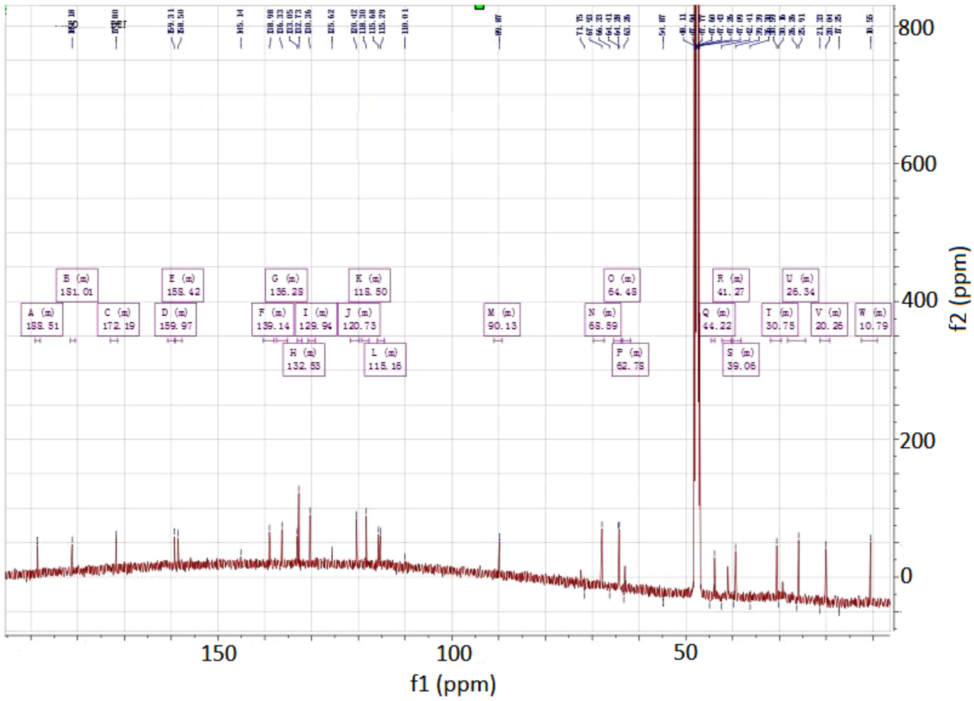

(2) ^{13}C NMR spectrum

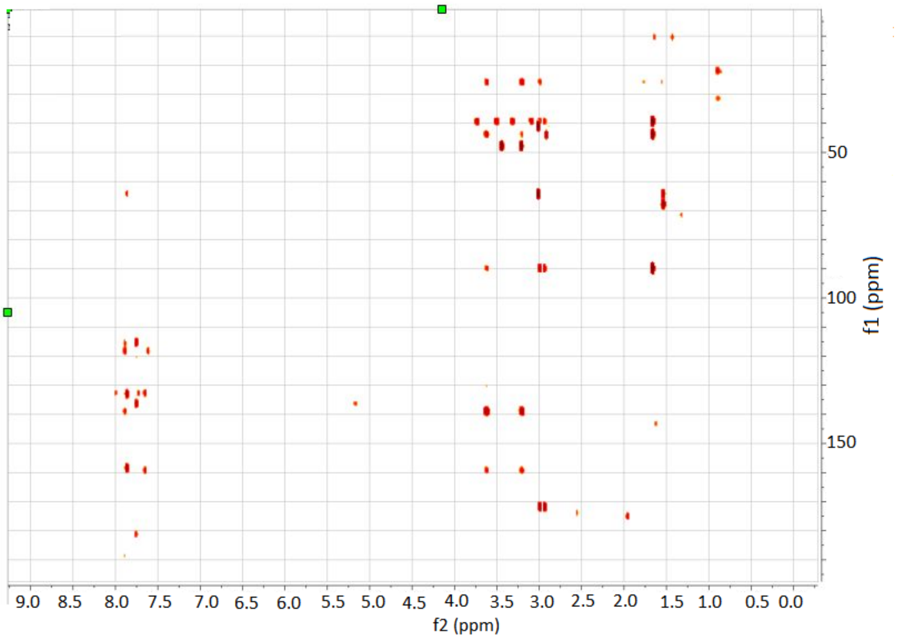

(3) HMBC spectrum
